# Establishment, functional dissection, and single-cell transcriptomal characteristics of patient-derived pulmonary sarcomatoid carcinoma organoids

**DOI:** 10.3389/fonc.2026.1867600

**Published:** 2026-06-29

**Authors:** Chaoyi Jia, Chen Chen, Jinghao Liu, Hongfeng Wu, Yuming Gao, Hongbing Zhang, Zihe Zhang, Xuanguang Li, Yongwen Li, Hongyu Liu, Jun Chen

**Affiliations:** 1 Department of Lung Cancer Surgery, Center of Thoracic Surgery, Tianjin Medical University General Hospital, Tianjin, China; 2 Tianjin Key Laboratory of Lung Cancer Metastasis and Tumor Microenvironment, Tianjin Lung Cancer Institute, Tianjin Medical University General Hospital, Tianjin, China

**Keywords:** organoids, precision oncology, pulmonary sarcomatoid carcinoma (PSC), single-cell transcriptome profiling, tumor heterogeneity

## Abstract

**Background:**

Pulmonary sarcomatoid carcinoma (PSC) is a rare and highly aggressive subtype of non-small cell lung cancer with poor clinical outcomes. Progress in PSC research has been hindered by the lack of reliable preclinical models that faithfully recapitulate tumor heterogeneity and biological behavior. This study aimed to establish and characterize patient-derived organoid (PDO) models of PSC and evaluate their translational potential.

**Methods:**

Tumor tissues obtained from a surgically resected PSC patient were used to generate organoid cultures. Histopathological fidelity was assessed by hematoxylin and eosin staining and immunohistochemistry for CK, CK7, Vimentin, and BRG1. Tumorigenicity was evaluated using NOD-SCID gamma mouse xenograft models. Drug sensitivity to cisplatin and docetaxel was tested using ATP-based viability assays. Single-cell RNA sequencing was performed on matched primary tumors and organoids to compare cellular composition, transcriptomic profiles, and copy number variation (CNV) patterns.

**Results:**

Two stable PSC organoid lines (PDO1 and PDO2) were successfully established and maintained long-term in culture. Both organoids preserved the characteristic biphasic phenotype of PSC, showing co-expression of epithelial and mesenchymal markers. Xenograft experiments demonstrated robust tumorigenicity, with a 100% tumor formation rate, and xenograft tumors retained the histological architecture of the original tumor. Drug testing revealed heterogeneous chemosensitivity between the two organoid lines, with PDO1 showing greater sensitivity to cisplatin and docetaxel than PDO2. Single-cell transcriptomic analysis demonstrated that the organoids largely retained the malignant cellular populations, transcriptomic signatures, and CNV landscapes of the matched primary tumors. Correlation analysis of highly variable genes showed strong similarity between PDOs and their parental tumors (r = 0.7–0.8). Module score analysis revealed increased epithelial features and reduced mesenchymal features in organoids compared with matched primary tumors, indicating partial EMT state remodeling during *in vitro* culture. Pseudotime analysis further supported a branched EMT-associated transition from KRT-high epithelial carcinoma-like states toward VIM-high sarcomatoid-like states.

**Conclusion:**

We established and validated two PSC patient-derived organoid models that faithfully recapitulate key pathological, genomic, and functional features of the original tumors. These models provide a valuable platform for studying PSC biology, investigating tumor heterogeneity, and screening personalized therapeutic strategies for this rare and aggressive malignancy.

## Introduction

Pulmonary sarcomatoid carcinoma (PSC) is a distinct subtype of lung cancer characterized by high invasiveness and poor prognosis, accounting for approximately 0.5% of non-small cell lung cancer (NSCLC) cases (1).According to the classification of the World Health Organization (WHO), PSC is divided into three major categories: carcinosarcoma (CS), pulmonary blastoma (PB), and pleomorphic carcinoma (PCC) (2). The definitive diagnosis of PSC relies on histological features, including the coexistence of both carcinomatous and sarcomatous components within the tumor (3). Hematoxylin and eosin (H&E) staining reveals a mixed growth pattern consisting of conventional lung cancer components (e.g., adenocarcinoma or squamous cell carcinoma) and sarcomatous component. The key diagnostic criterion relies on immunohistochemistry (IHC). Taking the most common clinical subtype, PCC, as an example, IHC demonstrates the co-expression of epithelial markers (e.g., CK, CK7) and mesenchymal markers (e.g., vimentin) within the sarcomatoid component (4).This dual-lineage immunophenotype indicates the epithelial origin of the sarcomatous components in PSC, thereby differentiating PSC from other mesenchymal sarcomas.

Surgery remains a reliable therapeutic option for early-stage resectable PSC. However, most patients present with advanced disease at diagnosis, rendering them ineligible for surgical resection (5). Additionally, patients exhibit a high postoperative recurrence rate (6, 7). Conventional radiotherapy and chemotherapy demonstrate limited efficacy in PSC treatment (8, 9). All these factors contribute to a worse prognosis in PSC patients compared with other NSCLC subtypes. Recent studies suggest that PSC exhibits higher tumor mutational burden (TMB) (10) and programmed death-ligand 1 (PD-L1) expression (4, 11, 12) than other NSCLC subtypes, indicating the potential of immunotherapy to improve patient outcomes. This hypothesis has been supported by case reports (13–16). However, such evidence remains preliminary and lacks validation from large prospective clinical trials.

Regarding mechanistic investigations, emerging evidence highlights the critical role of epithelial–mesenchymal transition (EMT) in PSC tumorigenesis and progression (3, 17). A high frequency of MET mutations, particularly MET exon 14 skipping mutations occurred in PSCs, suggests that this gene may serve as a potential therapeutic target (18–20). However, research into the molecular mechanisms underlying PSC has progressed slowly, lagging far behind that of other NSCLC subtypes. This is largely due to the historical lack of stable and reliable preclinical PSC models for experimental research, which obstructs the development of precise and effective therapeutic strategies to improve patient outcomes. Furthermore, owing to the difficulty in obtaining clinical samples, studies exploring the transcriptome and genetic characteristics of PSC remain limited.

Patient-derived organoids (PDOs) are three-dimensional (3D) miniature organ models cultured *in vitro* from patient tissue or cell samples (e.g., tumor tissues, body fluid-derived cells) (21). These models faithfully recapitulate the genetic and histopathological features of the original tumors and enable long-term passaging and maintenance, thereby supporting *in vitro* and *in vivo* drug sensitivity assays and tumorigenicity studies to characterize tumor biology (22). Furthermore, PDOs provide a robust platform for elucidating the molecular mechanisms underlying tumor progression (23, 24).

In this study, we describe the establishment of PDOs from tumor tissues of PSC patient and evaluate their biological characteristics using pathological examination, *in vivo* tumorigenicity assays, and *in vitro* drug sensitivity testing. We also perform single-cell RNA sequencing on the PDOs and their matched primary tumors to characterize their cellular composition and molecular features. Our results demonstrate that these PDOs faithfully recapitulate the molecular characteristics of the corresponding primary tumors, indicating that such organoids represent a valuable research models which may serve as a promising preclinical tool to dissect the biological behaviors of PSC, screen potential therapeutic agents, and facilitate the development of personalized treatment strategies for this aggressive malignancy.

## Materials and methods

### Specimens from PSC patient and ethics approval

The patient was diagnosed with NSCLC with sarcomatoid components by biopsy at the Department of Lung Cancer Surgery, Tianjin Medical University General Hospital (TMUGH). Clinical targeted next-generation sequencing identified a *KRAS* exon 2 missense mutation (c.37G>T, p.G13C) in the tumor tissue. Subsequently, the patient received four cycles of neoadjuvant therapy comprising nab-paclitaxel, platinum-based chemotherapy, adebrelimab, and apatinib prior to surgery, followed by surgical resection in the same department. Pathological diagnosis was confirmed by board-certified pathologists using the surgical specimens, and the tumor was diagnosed as PSC of the PCC type, with histological components consisting of 10% poorly differentiated adenocarcinoma and 90% sarcomatoid carcinoma. Written informed consent was obtained from the patient before specimen collection. This study was approved by the Clinical Research Ethics Review Committee of Tianjin Medical University General Hospital (Ethics Approval No.: IRB2024-YX-045-01) and was conducted in strict accordance with the ethical principles outlined in the Declaration of Helsinki.

### Tumor organoid culture

Fresh pulmonary sarcomatoid carcinoma tissue obtained from the resection was placed into a 1.5 mL Eppendorf tube and rinsed three times with HBSS supplemented with penicillin/streptomycin (P/S). The tissue specimen was minced with surgical scissors and transferred to a centrifuge tube. Three milliliters of digestion solution (DMEM/F12 medium containing 1% penicillin/streptomycin/amphotericin B, 1 mg/mL collagenase/dispase, and 0.001% DNase I) was added, and the tube was placed horizontally on a shaker (70 rpm, 37 °C) for 20 min. After settling, the supernatant was transferred to a new centrifuge tube, and 3 mL of HBSS was added to terminate digestion. The remaining tissue was incubated with an additional 3 mL of digestion solution for 15–20 min under the same conditions, followed by digestion termination with 3 mL of HBSS. Both digested suspensions were filtered through a 70 μm cell strainer, pooled, and centrifuged at 1200 × g for 5 min. The cell pellet was resuspended in 1 mL of DPBS. Following centrifugation at 300 × g for 5 min, the supernatant was discarded, and the cell pellet was resuspended in Matrigel (Corning, NY, USA) at a density of 2–4 × 10³ cells/μL at a 1:2 ratio (culture medium:Matrigel). The Matrigel-cell suspension was seeded into pre-warmed (37 °C) culture plates and incubated at 37 °C for 5 min. The plates were then inverted and left undisturbed for 25 min to allow Matrigel solidification. Six hundred microliters of pre-warmed MasterAim Tumor Organoid Complete Medium (AimingMed, Hangzhou, China) was gently added to each well along the well walls using a pipette. Cultures were maintained at 37 °C in an incubator with 5% CO_2_.

### Xenografts

All animal experiments strictly adhered to the Animal Protection Law and were approved by the Institutional and Governmental Animal Welfare Committee prior to initiation. NOD SCID gamma (NSG; NOD.Cg-Prkdc^scid^ Il2rg^tm1Wjl/SzJ^) mice were purchased from GemPharmatech LLC (Jiangsu, China). The NSG mice were housed in the Laboratory Animal Center of the Institute of Hematology, Chinese Academy of Medical Sciences, under specific pathogen-free (SPF) conditions, with a 12-h light/dark cycle, and provided with ad libitum access to standard laboratory chow and water. For xenograft model establishment, the eighth passage (P8) organoids were used. Twenty intact organoid spheroids from each organoid line were harvested and resuspended in a 1:1 mixture of organoid culture medium and Matrigel. The organoids were subcutaneously injected into the hind limbs of NSG mice at a volume of 100 μL per mouse, corresponding to approximately 1 × 10^6^ cells. Subcutaneous tumor growth was monitored via caliper measurements, and tumor volume was calculated using the following formula: V = (width² × length)/2, where width represents the smaller tumor dimension and length denotes the larger dimension. Tumor doubling time (DT) was calculated using the following formula: DT = (t_2_ – t_1_) × log (2)/log(V_2_/V_1_), where t_1_ and t_2_ represent the initial and final time points, and V_1_ and V_2_ denote the corresponding tumor volumes.The Committee of Animal Care and Use of Tianjin Medical University General Hospital approved all experiments with animals in this study (No. IRB2022-KY-183) and were performed in accordance with the”China Guide for the Protection and Use of Laboratory Animals”.

### *In vitro* drug treatment assays

After digestion and centrifugation, the nineth passage (P9) organoids were transferred to 96-well plates at a density of 8, 000–10, 000 cells per well, mixed with 10 μL of Matrigel, and evenly seeded. Subsequently, 200 μL of culture medium was added to each well. Drug treatment was initiated after 72 h of culture using cisplatin or docetaxel. Following 72 h of drug exposure, organoid viability was assessed using the MasterAim Organoid Viability ATP Assay Kit (AimingMed, Hangzhou, China). The detection reagent was mixed with culture medium at a 1:1 ratio, and 200 μL of the mixture was added to each well. The plates were subjected to vigorous linear shaking for 2 min at room temperature using a plate reader to ensure complete cell lysis. The plates were then incubated for 8 min to allow the luminescent signal to stabilize, and the luminescence intensity was recorded using an EnSight Multimode Plate Reader (Revvity, Shanghai, China).

### Histology and IHC

All histological procedures were performed in accordance with standard protocols. Primary tumors, the third passage (P3) organoids, and xenograft tumors were fixed in 4% paraformaldehyde (PFA), paraffin-embedded, and sectioned into 4 μm slices. Tissue sections were processed by Wuhan Servicebio Technology Co., Ltd. for both hematoxylin and eosin (H&E) staining and IHC analysis using antibodies against Cytokeratin (CK), Cytokeratin 7 (CK7), Vimentin (VIM), and Brahma-related gene 1 (BRG1). After staining, slides were digitally scanned using the SQD-12P Slide Scanning System (Shenzhen Shengqiang Technology Co., Ltd., Shenzhen, China).

### Tissue dissociation and cell purification for single-cell sequencing

Freshly resected primary tumor and the forth passage (P4) organoids specimens were preserved in ice-cold tissue preservation solution and transported to Shanghai Hongxu Biotechnology Co., Ltd., within 2 h. Under sterile conditions, the specimens were washed twice with pre-chilled RPMI 1640 medium supplemented with 0.04% bovine serum albumin (BSA). The tissues were minced into ~0.5 mm³ fragments using surgical scissors and incubated in freshly prepared digestion solution containing RPMI 1640, 0.04% BSA, and 0.2% collagenase I at 37 °C for 30–60 min, with inversion mixing every 5–10 min. The digested cell suspension was filtered through a BD 40 μm cell strainer once or twice, followed by centrifugation at 300 × g for 5 min at 4 °C. The pellet was resuspended in an appropriate volume of culture medium and mixed with an equal volume of red blood cell lysis buffer. Next, the mixture was incubated on ice for 10 min, centrifuged at 300 × g for 5 min, and the supernatant was discarded. The pellet was washed once with culture medium under the same centrifugation conditions and then resuspended in 100 μL of RPMI 1640 supplemented with 0.04% BSA. Cell concentration and viability of the single-cell suspension were determined using a Luna-FL™ cell counter or trypan blue staining.

### Single-cell library construction and transcriptomal sequencing

Freshly prepared single-cell suspensions were adjusted to a cell concentration of 700–1, 200 cells/μL. According to the manufacturer’s instructions for the MobiCube High-Throughput Single-Cell 3’ Transcriptome Kit V2.1 (Cat. #PN-S050200301; Jiaxing, China), cell loading and library construction were performed on the MobiNova-100 microfluidic platform (ZesoStar Technologies, Wuhan, China). The resulting libraries were sequenced on an Illumina NovaSeq 6000 PE150 platform. Raw sequencing data were aligned to the human reference genome (GRCh38) using MobiVision (version 3.2), the official quality control software from ZesoStar, with default parameters. Cell filtering and gene quantification were performed to generate a feature-barcode matrix for downstream analysis. This matrix was imported into R software and further processed using the Seurat R package (v5.2.1). Low-quality cells were filtered based on the following criteria: fewer than 500 detected genes per cell, mitochondrial gene content exceeding 30%, or fewer than 1, 000 unique transcripts per cell. Additionally, genes expressed in fewer than 10 cells were excluded. Gene expression levels were normalized using the LogNormalize function in Seurat. A total of 2, 000 highly variable genes were selected for dimensionality reduction via principal component analysis (PCA), and subsequent nonlinear dimensionality reduction was performed using t-distributed stochastic neighbor embedding (t-SNE).

### Single-cell copy number variation inference

Somatic large-scale chromosomal copy number variations (CNVs) were inferred using the *inferCNV* R package (v1.22.0) (25). Briefly, a new gene-cell matrix was generated incorporating epithelial cells and sarcomatoid cells, annotation data, and gene/chromosomal position files. T cells and B cells, which are assumed to lack CNVs, served as reference populations. CNV scores for each cluster were calculated as the sum of squared values across CNV regions.

### Pseudotime trajectory analysis

Pseudotime trajectory analysis was performed using the Monocle3 R package. CNV-supported malignant epithelial and sarcomatoid populations were extracted from the Seurat object and used for trajectory construction. UMAP embeddings were used for dimensionality reduction and trajectory visualization. Expression changes of representative epithelial and mesenchymal markers along pseudotime were visualized using ggplot2.

### Statistics

All statistical analyses were performed using GraphPad Prism (version 10.1.2; GraphPad Software, San Diego, CA, USA) and R software (version 4.4.3). For continuous variables, independent-samples t-test or Mann–Whitney U test was used to compare differences between two groups. For categorical variables, the chi-square test was performed. Statistical significance was defined as P < 0.05.

## Result

### Establishment of human PSC organoids from surgical specimens

**Figure 1:**
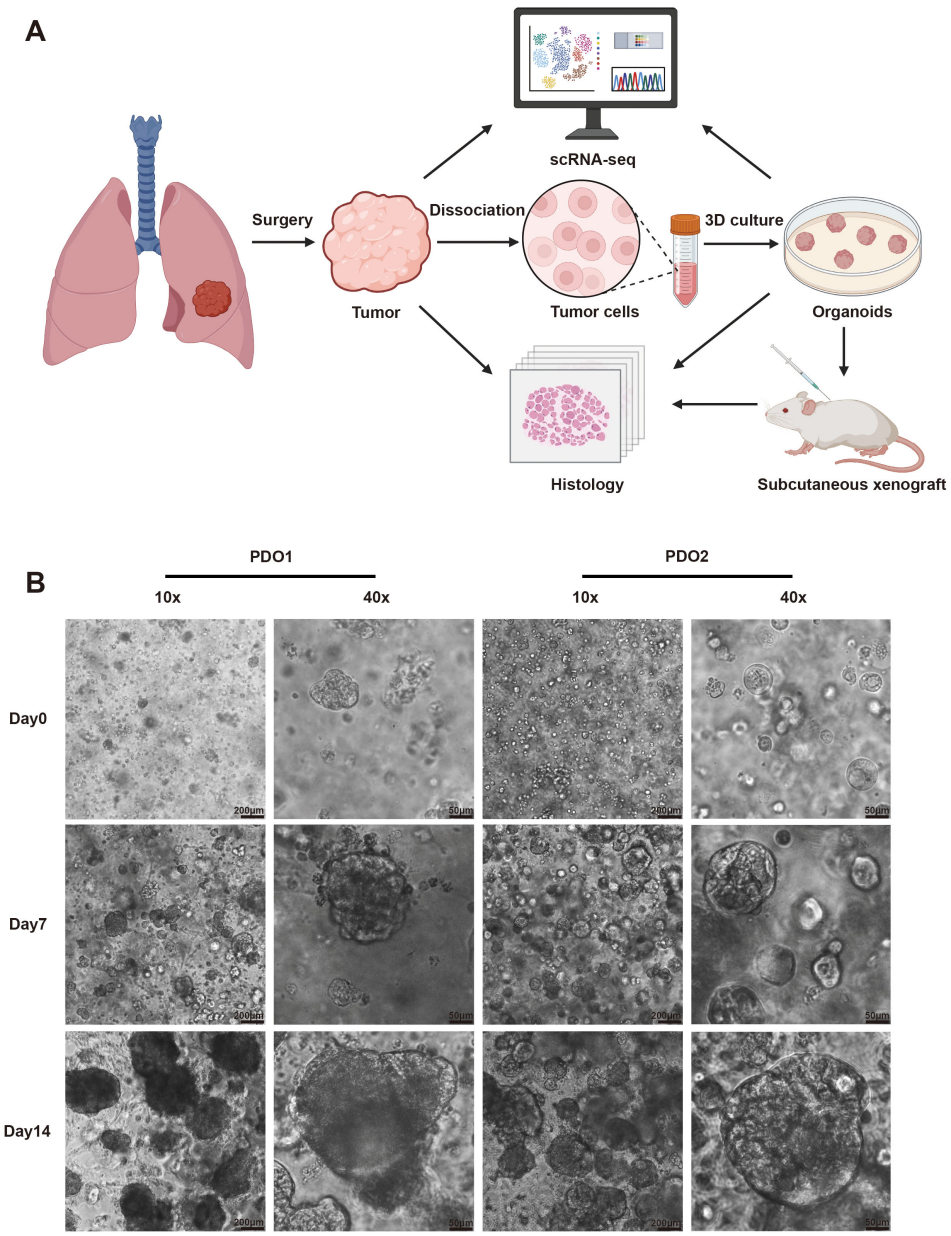
Flowchart and microscopic organoids. **(A)** Research flowchart. **(B)** Microscopic morphological changes of the two organoids over time.

To establish PSC organoids, we collected tumor tissues from the right upper lobe tumor of a patient who was diagnosed with NSCLC with sarcomatoid components by biopsy and had received neoadjuvant therapy comprising chemotherapy (nab-paclitaxel + platinum), immunotherapy (adebrelimab), and anti-angiogenic therapy (apatinib); the treatment response was assessed as stable disease (SD) prior to surgery. Clinical targeted next-generation sequencing (NGS) identified a *KRAS* exon 2 missense mutation (c.37G>T, p.G13C) in the tumor tissue, whereas no *MET* exon 14 skipping alteration or other currently actionable driver mutations were detected. The patient underwent resection of the right upper lobe of the lung and lymph node dissection at the Department of Lung Cancer Surgery, TMUGH, and tumor tissues were obtained immediately after resection. Three distinct sites of the tumor tissues were collected for PDO culture and single-cell sequencing. Given the current lack of a dedicated organoid culture system for PSC, we utilized the lung adenocarcinoma organoid culture system based on the patient’s previous clinical diagnosis, which included adenocarcinoma components. Of the three tumor tissue samples obtained, two grew and were maintained for stable passaging; these were designated as organoids PDO1 and PDO2, respectively. Morphologically, both PDO1 and PDO2 grew in clusters, with cellular aggregates gradually increasing in size over time and culture doubling times of 10–14 days (**Figure 1B**).

### Patient-derived PSC organoids retained the IHC features of the primary tumor

**Figure 2:**
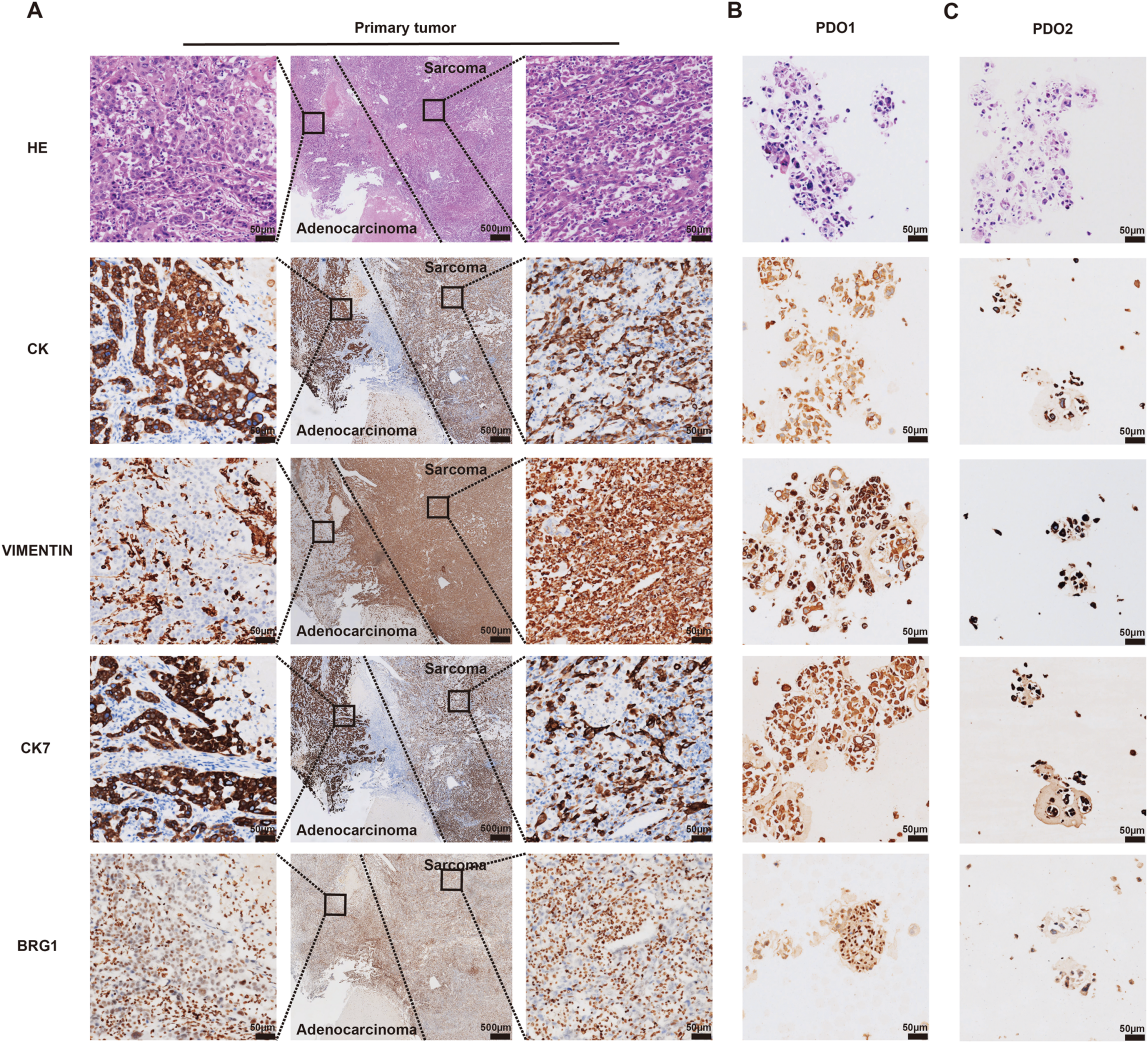
H&E staining and IHC staining for primary tumor and organoids of PSCs.H&E staining and IHC staining result of **(A)** Primary tumor, **(B)** PDO1 and **(C)** PDO2.

Pathological features of the primary tumor of this PSC patient, which revealed by H&E staining and IHC staining (**Figure 2A**) visually demonstrated a mixed growth pattern of carcinoma and sarcoma components. In detail, the primary tumor comprised a small proportion of poorly differentiated adenocarcinoma (10%) and a predominant sarcomatoid carcinoma component (90%). Notably, both components exhibited positivity for the epithelial markers CK and CK7, as well as the mesenchymal markers Vimentin (VIM) and BRG1. H&E staining of the PDOs (**Figures 2B, C**) showed both of which exhibited clustered growth patterns and no significant morphological differences between PDO1 and PDO2. IHC analysis revealed that both PDOs co-expressed CK and VIM, indicating that the PDOs retained both epithelial and mesenchymal characteristics (**Figures 2B, C**). This dual-lineage phenotype represents one of the most distinctive hallmarks of PSC.

### Tumorigenic potential of the PDOs in mouse xenograft models

**Figure 3:**
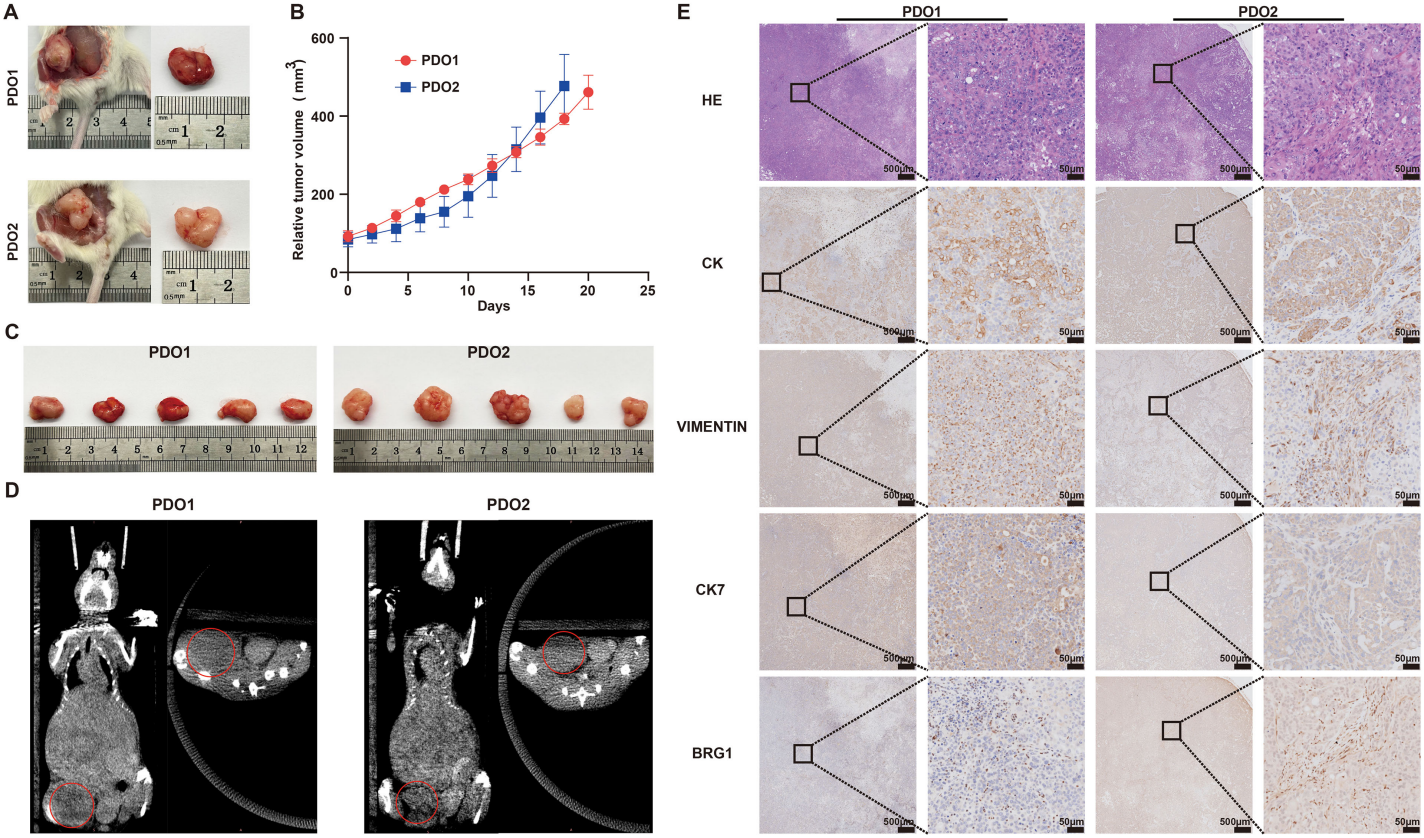
Tumorigenic potential of the PDOs in mouse xenograft models. **(A, C)** Representative figures of subcutaneous tumors derived from PDO1 and PDO2 organoid lines at mouse sacrifice. **(B)** Growth curves of subcutaneous tumors from the two organoid lines. **(D)** CT imaging of subcutaneous tumors derived from the two organoid lines. **(E)** H&E staining and immunohistochemical results.

Subsequently, we evaluated the tumorigenic potential of the PDOs in mouse xenograft models. The two organoid lines were subcutaneously injected into immunodeficient NSG mice. The results showed that both PDO1 (5/5) and PDO2 (5/5) exhibited 100% tumor formation efficiency. The formation of subcutaneous xenograft tumors was confirmed by CT (**Figure 3D**) and autopsy (**Figures 2A, C**). The tumor doubling times of PDO1 and PDO2 were approximately 8.69 ± 1.06 days and 7.87 ± 0.59 days, respectively (**Figure 3B**). Histological staining demonstrated that the histological features of the mouse xenografts (**Figure 3E**) were consistent with those of the primary tumor and organoids (**Figure 2**), with IHC confirmed the positive expression of the aforementioned markers, which was consistent with their expression status in the primary tumor and organoids (**Figure 3E**).

### *In vitro* chemotherapeutic drug sensitivity of PDOs

**Figure 4:**
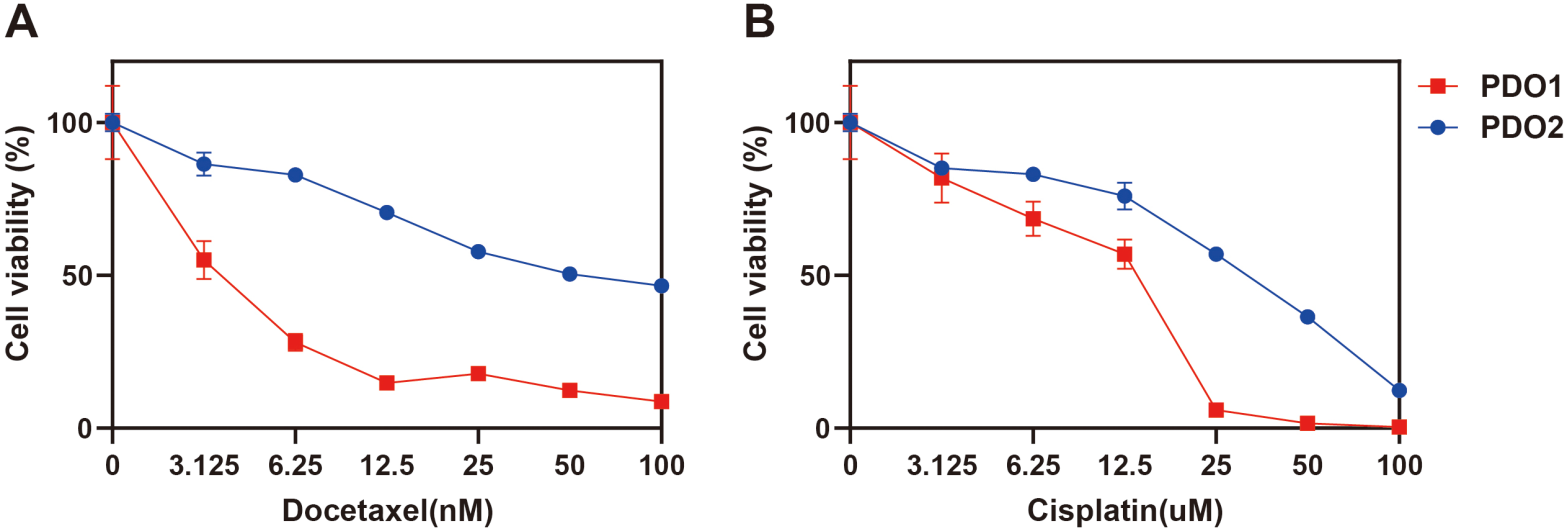
*In vitro* chemotherapeutic drug sensitivity of PDOs. **(A)** Dose-response curve of PDOs to docetaxel. **(B)** Dose-response curve of PDOs to cisplatin.

In clinical practice, cisplatin and docetaxel are commonly used chemotherapeutic agents for PSC. To evaluate the therapeutic responses of PDO1 and PDO2 to these conventional chemotherapeutic agents, *in vitro* drug sensitivity assays were performed using cisplatin and docetaxel in the two PDO models with Organoid Viability ATP Assays. The IC_50_ values for cisplatin were 10.66 μM (95% CI: 8.73–12.86) for PDO1 and 28.78 μM (95% CI: 25.30–32.74) for PDO2. For docetaxel, the IC_50_ values were 2.962 nM (95% CI: 1.93–3.88) for PDO1 and 59.26 nM (95% CI: 49.73–72.83) for PDO2. The two PDOs exhibited distinct responses to the same chemotherapeutic agents, with PDO1 showing higher sensitivity to standard chemotherapy compared with PDO2 (**Figure 4**).

### Cellular landscape of primary tumors and organoids revealed by single-cell sequencing

**Figure 5:**
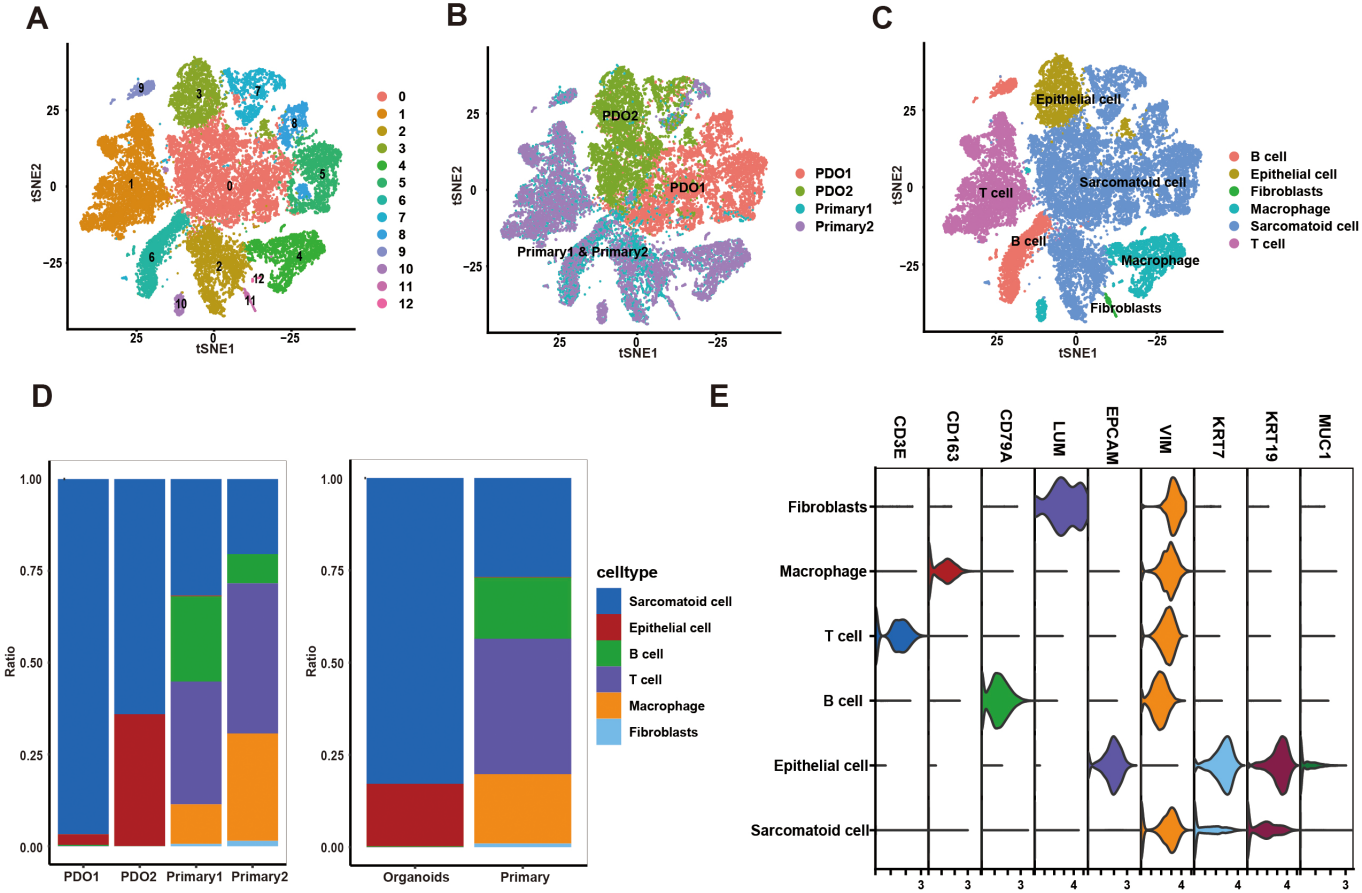
Single-cell RNA sequencing (scRNA-seq) reveals the cellular composition of primary tumors and organoids. **(A–C)** t-SNE plots depicting original cell clusters, sample annotations, and reclassified cell types. **(D)** Box plots illustrating proportions of distinct cell types across samples. **(E)** Violin plots displaying normalized expression levels of established marker genes for different cell types.

To compare the gene expression profiles of the primary tumors and organoids, single-cell transcriptome sequencing was performed on both the primary tumor tissues and the subsequently cultured organoids. After stringent quality control, a gene-cell matrix containing 31 919 cells and 29 241 genes was constructed. Using t-distributed stochastic neighbor embedding (t-SNE) analysis, cells were grouped into distinct clusters in two-dimensional space based on their transcriptional profile characteristics. These clusters were annotated as known cell types, including epithelial cells (*EPCAM^+^*), fibroblasts (*LUM^+^*), T cells (*CD3E^+^*), macrophages (*CD163^+^*), and B cells (*CD79A^+^*). Since no specific cellular markers for sarcomatoid carcinoma components have been reported in previous studies, we used the co-expression of the mesenchymal marker *VIM* and the epithelial markers *KRT7* (CK7) and *KRT19* (CK19) as identification criteria, consistent with the conventional clinicopathological diagnostic markers for PSC (**Figure 5E**). The two primary tumor samples exhibited similar cellular compositions, including sarcomatoid cells, epithelial cells, T cells, B cells, macrophages, and fibroblasts; Primary Tumor 1 showed a higher proportion of sarcomatoid cells, while Primary Tumor 2 was enriched in macrophages. However, both organoid lines consisted almost entirely of epithelial and sarcomatoid cells: PDO1 was composed almost entirely of sarcomatoid cells, whereas PDO2 contained a subset of epithelial cells (**Figures 5B–D**).

### The organoids largely retained the CNV patterns of the primary tumor

**Figure 6:**
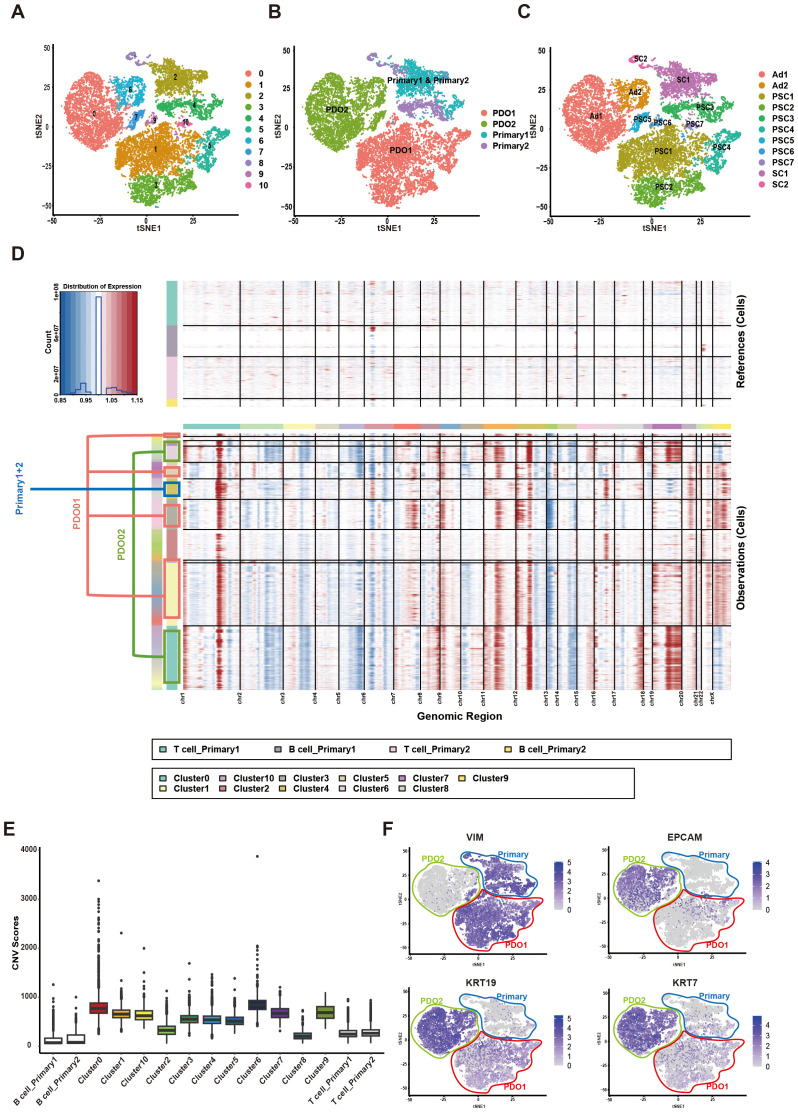
Organoids retain the CNV patterns of the primary tumor. **(A–C)** t-SNE plots illustrating cell subpopulations, sample annotations, and reclassified cell types. **(D)** Heatmap depicting genome-wide CNV profiles across epithelial cell subpopulations. Blue and red indicate low and high CNV levels, respectively. T cells and B cells were used as reference populations. Regions of interest in CNVs are highlighted with distinct-colored borders. **(F)** Expression of *VIM*, *EPCAM, KRT18*, and *KRT7* superimposed on t-SNE plots. Color intensity (ranging from white to purple) represents the average expression levels of these genes.

To further compare the CNV patterns of primary tumor cells and organoid cells, we extracted cells previously annotated as epithelial cells and sarcomatoid cells and reclassified them into 11 clusters (**Figure 6A**). Since immune cells are generally considered to lack CNVs, we performed InferCNV analysis using T and B cells from the primary tumor as references to calculate the CNV score for each cluster. Except for clusters 2 and 8, the CNV scores of the other clusters were significantly higher than those of immune cells (**Figure 6E**). The detailed CNV score values for each cluster are provided in **Supplementary Table 2**. Therefore, clusters other than clusters 2 and 8 were designated as malignant cells. Based on distinct marker expression patterns (**Figure 6F**), these malignant cells were further classified into carcinoma components (adenocarcinoma, Ad1 and Ad2) and sarcomatoid components (pulmonary sarcomatoid carcinoma, PSC1–7) (**Figure 6C**). Notably, the CNV patterns of the primary tumor were largely retained in the organoids: copy number gains in chr1, chr12, chr20, and chrX, as well as copy number loss in chr6, were consistent between the organoids and primary tumors(**Figure 6D**). However, the organoids also exhibited some unique CNV patterns absent in the primary tumor: PDO1 harbored additional CNVs in chr6, chr11, and chr13, whereas PDO2 harbored unique CNVs in chr7, chr9, and chr11 that were not present in the primary tumor.

### PDOs recapitulated the transcriptomic characteristics of the primary tumors

**Figure 7:**
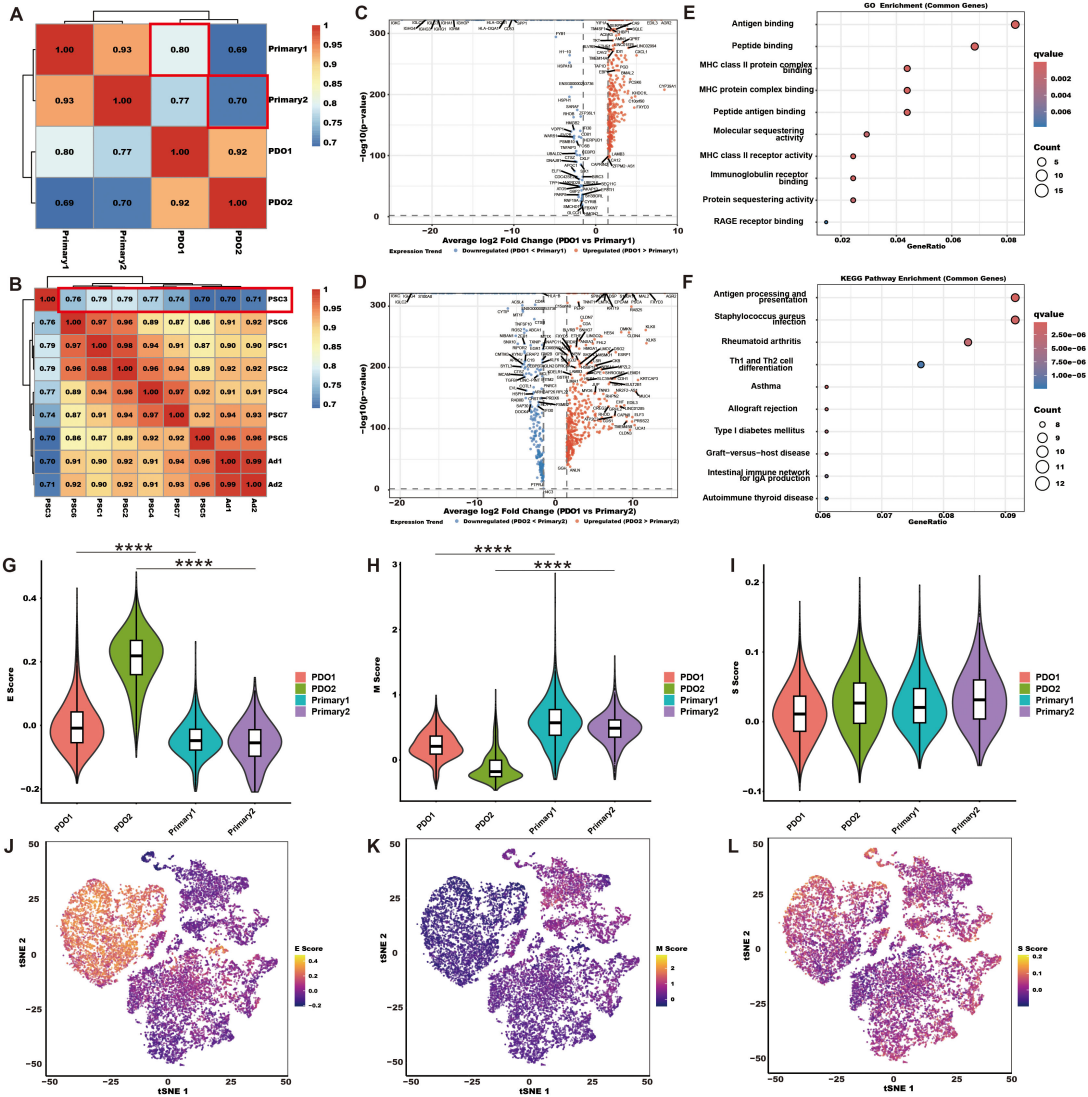
PDOs recapitulated the transcriptomic characteristics of the primary tumors. **(A, B)** Comparative correlation analysis of the top 1 000 highly variable genes among primary tumors, PDOs and tumor cell clusters. **(C, D)** Volcano plots illustrating differentially expressed genes (DEGs) between organoids and their matched primary tumors. Red and blue dots represent upregulated and downregulated genes, respectively. **(E, F)** GO and KEGG pathway enrichment analyses of DEGs that overlapped between the paired comparisons. **(G–I)** Violin plots comparing Epithelial score (E score), Mesenchymal score (M score), and Cancer Stem Cell score (S score) between organoids and matched primary tumors. **(J–L)** Visualization of these scores superimposed on tSNE dimensionality reduction and clustering plots.

We compared the transcriptomic profiles of epithelial cells and sarcomatoid cells between primary tumors and their matched organoids. The top 1000 highly variable genes (HVGs) were compared between primary tumors and matched PDOs. Correlation analysis of the HVGs between organoids and primary tumors revealed that the correlation coefficient between PDO1 and Primary1 was 0.8, and that between PDO2 and Primary2 was 0.7 (**Figure 7A**). Notably, the correlation coefficients between tumor cells from primary tumors and organoids exceeded 0.7 (**Figure 7B**). Meanwhile, differentially expressed genes (DEGs) between the primary tumors and PDOs were identified using a logFC threshold of 1.5 (**Figures 7C, D**). Using PDO samples as a reference, two sets of DEGs were identified through comparison with the corresponding primary tumor tissues. Both DEG sets comprised a substantial number of genes. To elucidate the impact of organoid culture on epithelial cells and sarcomatoid cells in PSC, an intersection analysis was performed to identify common DEGs between the two datasets. Functional enrichment analysis using GO and KEGG revealed that these common DEGs were significantly enriched in antigen presentation–related pathways.(**Figures 7E, F**). Subsequently, the ‘AddModuleScore’ function was used to calculate the Epithelial score (E score), Mesenchymal score (M score), and Cancer Stem Cell score (S score) for each sample using published gene signatures (**Supplementary Table 1**). In the paired comparison, PDOs exhibited higher E scores, lower M scores, and no significant difference in S scores compared with primary tumors (**Figures 7G–L**).

### Pseudotime analysis revealed an EMT-associated transition in PSC malignant cells

**Figure 8:**
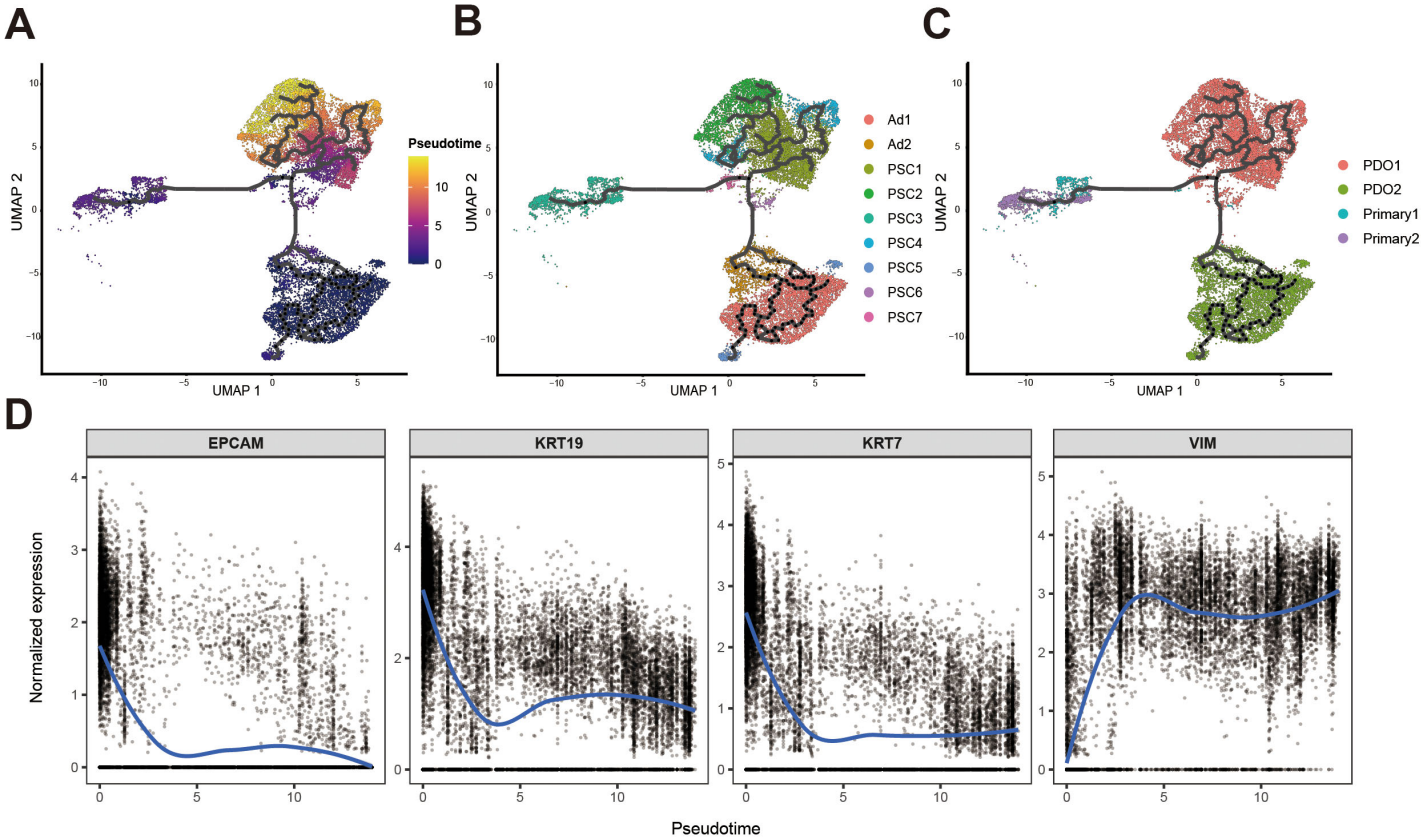
Pseudotime trajectory analysis reveals EMT-associated transcriptional transition in PSC malignant cells. **(A)** Monocle3 pseudotime trajectory analysis of CNV-supported malignant epithelial and sarcomatoid populations. Cells are colored by pseudotime. **(B)** Pseudotime trajectory colored by tumor cell annotations, including epithelial carcinoma-like populations and sarcomatoid-like PSC populations. **(C)** Pseudotime trajectory colored by sample origin, including primary tumor samples and PDO lines. **(D)** Expression dynamics of representative epithelial and mesenchymal markers along pseudotime. *EPCAM, KRT19*, and *KRT7* showed higher expression in early pseudotime states and decreased along the trajectory, whereas *VIM* increased along pseudotime, supporting an EMT-associated transition from epithelial carcinoma-like states toward sarcomatoid-like malignant states.

To further investigate whether epithelial carcinoma-like and sarcomatoid-like malignant populations were transcriptionally connected through an EMT-associated transition, we performed pseudotime trajectory analysis using CNV-supported malignant cells. Based on their KRT-high epithelial carcinoma-like features, Ad1 and Ad2 were selected as the root populations for pseudotime ordering. The trajectory analysis revealed a branched evolutionary structure rather than a strictly linear path (**Figure 8A**). When colored by tumor cell annotation, epithelial carcinoma-like populations, including Ad1 and Ad2, and sarcomatoid-like populations, including PSC1–PSC7, were distributed along interconnected but divergent branches. Notably, several sarcomatoid-like subpopulations, particularly PSC1, PSC2, and PSC4, were enriched in branch-associated or terminal trajectory regions, suggesting that they may represent relatively late sarcomatoid differentiation states, whereas PSC6 and PSC7 were located closer to trajectory junctions and may reflect intermediate EMT-associated transitional states (**Figures 8B, C**). Epithelial markers, including *EPCAM*, *KRT19*, and *KRT7*, showed relatively high expression at early pseudotime states and gradually decreased along the trajectory. In contrast, the mesenchymal marker *VIM* increased markedly with pseudotime and remained elevated in later states (**Figure 8D**).

## Discussion

PSC is a rare and highly aggressive subtype of NSCLC. However, the lack of stable preclinical models has severely hampered research into the molecular mechanisms underlying PSC pathogenesis and the screening of potential therapeutic agents for this disease, leaving such research significantly lagging behind that on other NSCLC subtypes.

To address this gap and accelerate progress in PSC-related research, in the present study we successfully established two stable PDO models derived from PSC patient. Histological staining confirmed that the established PDOs recapitulated the histological features of the primary tumors, notably the co-expression of epithelial and mesenchymal markers in tumor tissues—a hallmark that serves as the diagnostic criterion for this rare tumor type. *In vitro* drug sensitivity assays demonstrated that the PDOs were responsive to docetaxel and cisplatin, the standard chemotherapeutic agents for this tumor type. These organoids exhibited robust tumorigenicity in immunodeficient mice, with a 100% tumor formation rate, and histological staining of the xenograft tumors further verified that they retained the histological characteristics of the primary tumors. More importantly, we performed comparative single-cell transcriptomic analyses of PDOs and their matched primary tumor tissues. We compared the transcriptomic profiles of epithelial cells and sarcomatoid cells between primary tumors and their matched organoids. Furthermore, correlation analysis of the top 1 000 HVGs showed a high degree of correlation between the PDOs and primary tumor samples, as well as between the tumor cell clusters of PDOs and those of the primary tumors. Those results confirmed that these PDOs highly retained the transcriptomic features of the primary tumors. InferCNV analysis also revealed that the PDOs largely preserved the CNV patterns of the primary tumor tissues. Collectively, these findings demonstrate that the PDOs established in this study faithfully recapitulated the key characteristics of the primary tumors. Moreover, they are amenable to long-term stable culture and applicable for both *in vitro* and *in vivo* assays, thus representing a valid and qualified preclinical model for PSC research.

Meanwhile, we also observed several discrepancies between the two PDO lines. *In vitro* drug sensitivity assays indicated that PDO2 exhibited higher drug resistance than PDO1. Combined with previous reports establishing tumor heterogeneity as a key mechanism underlying drug resistance (26–28), this differential drug response is likely attributable to the distinct cellular compositions of the two PDO lines: PDO1 consists almost entirely of pulmonary sarcomatoid carcinoma cells, whereas PDO2 comprises both adenocarcinoma and pulmonary sarcomatoid carcinoma cells, resulting in higher intratumoral heterogeneity and may enhance drug resistance. Given that PDO1 and PDO2 were derived from different spatial regions of the same right upper lobe of lung, the differences in CK, CK7, and Vimentin expression may partly reflect regional intratumoral heterogeneity and variation in the relative proportions of adenocarcinoma-like and sarcomatoid-like components. These region-specific phenotypic differences may also have contributed to the distinct drug-response profiles of the two organoid lines, as heterogeneous epithelial and sarcomatoid cellular compositions could influence intrinsic chemosensitivity and resistance. InferCNV analysis revealed that, while the PDOs largely retained the CNV profiles of the primary tumors, they also harbored unique CNV events not detected in the primary tumor tissues. This phenomenon is likely associated with the enrichment of tumor cells and the elimination of the normal cell background during PDO culture (24, 29). Although this may limit their application in investigating tumor–stroma interactions, the organoids exhibit higher tumor purity and are amenable to efficient *in vitro* expansion compared with primary tumors (30, 31). These properties render the organoid models highly suitable as *in vitro* and *in vivo* experimental platforms for antitumor drug testing and for helping guide clinical treatment decisions. In subsequent paired analyses, we also identified a large number of DEGs between the PDOs and primary tumor tissues, which were primarily enriched in pathways associated with antigen presentation and related biological functions. This indicates that PDO culture exerts a certain modulatory effect on transcriptomic profiles, which is also linked to the near absence of the normal cell background present in primary tumor tissues—a common phenomenon observed during *in vitro* culture of PDOs (32, 33). Given previous reports suggesting a critical role of EMT in PSC progression, we compared EMT and cancer stem cell (CSC) features between PDOs and primary tumors. The results revealed that PDOs exhibited higher E-scores than primary tumors; notably, PDO2 showed significantly elevated E scores compared with other samples. This may be attributable to the dominance of adenocarcinoma components within PDO2. Conversely, M scores were lower in PDOs, potentially due to the loss of the native tumor microenvironment during organoid culture, which may disrupt the EMT process (34, 35). However, S scores showed no significant differences. The inter-sample variability in epithelial and mesenchymal scores may reflect the impact of *in vitro* culture conditions on tumor transcriptomic profiles and indirectly underscores the biological significance of EMT in PSC development. Pseudotime trajectory analysis further supported EMT-associated transcriptional plasticity in PSC. CNV-supported malignant cells exhibited a branched trajectory rather than a strictly linear transition. Along pseudotime, epithelial markers including *EPCAM, KRT7*, and *KRT19* tended to decrease, whereas *VIM* increased, suggesting a potential transition from KRT-high epithelial carcinoma-like states toward VIM-high sarcomatoid-like malignant states. This finding further highlights the biological relevance of EMT in PSC and supports the utility of these PDO models for studying sarcomatoid transformation.

An important clinical context of this study is that the tumor tissue used for PDO establishment was obtained after four cycles of intensive neoadjuvant therapy. Therefore, these PDOs likely represent residual treatment-resistant tumor populations rather than treatment-naive PSC cells. Previous studies have shown that EMT is a central biological process in PSC evolution and contributes to its aggressive phenotype (17). In addition, therapeutic pressure can reshape tumor clonal architecture by selecting resistant subclones (36), while EMT has been closely associated with platinum resistance, immune escape, and treatment tolerance (37, 38). In this case, platinum-based chemotherapy may have selected for tumor cells with enhanced DNA-damage repair or tolerance capacity (39), while immune checkpoint blockade and anti-angiogenic therapy may have further reshaped the tumor ecosystem through immune-mediated selection, vascular remodeling, and hypoxia-associated stress (40). These combined selective pressures may have shaped the baseline EMT states of the established PDOs, altered their clonal architecture, and may contributed to the heterogeneous drug-response profiles observed between PDO1 and PDO2, particularly the increased chemoresistance of PDO2. Consistently, inferCNV analysis revealed both shared and organoid-specific CNV alterations, suggesting clonal evolution and selective expansion during organoid establishment. Although this treatment background introduces biological complexity, it may also enhance the translational relevance of these PDO models by recapitulating clinically relevant residual and therapy-resistant PSC states.

In addition, targeted NGS identified a *KRAS G13C* mutation without detectable *MET* exon 14 skipping or other currently actionable driver alterations. Given the limited availability of effective targeted therapies for G13C KRAS-mutant NSCLC, these PDOs may provide a useful platform for future studies exploring treatment and potential combination strategies in KRAS-mutant PSC.

This study has several limitations that should be acknowledged. First, although tumor tissues were sampled from three spatially distinct regions and successfully used to generate two independent PDO lines with divergent transcriptomic and drug-response characteristics, both organoid models were ultimately derived from a single PSC patient. The inability of single-patient-derived PDOs to fully capture the complex biological heterogeneity of PSC represents a key limitation of this study. Second, we did not compare PDOs at different culture durations and passages with primary tumors; thus, the impact of culture duration on transcriptomic changes cannot be evaluated. Additionally, although we demonstrated that the two established PDO lines are suitable for both *in vitro* and *in vivo* experiments and performed *in vitro* drug sensitivity assays for conventional chemotherapeutic agents, we did not evaluate immunotherapy, targeted therapy, or other treatment regimens.

Collectively, our study establishes and validates two patient-derived organoid models for PSC, which may serve as a promising preclinical tool to dissect the biological behaviors of PSC, screen potential therapeutic agents, and facilitate the development of personalized treatment strategies for this aggressive malignancy.

## Data Availability

The raw sequence data reported in this paper have been deposited in the Genome Sequence Archive for Human (GSA-Human) at the National Genomics Data Center, China National Center for Bioinformation/Beijing Institute of Genomics, Chinese Academy of Sciences, under accession number HRA018857, and are publicly accessible at https://ngdc.cncb.ac.cn/search/specific?db=hra&q=HRA018857.
